# Is Preoperative Chemoradiotherapy Beneficial for Sphincter Preservation in Low-Lying Rectal Cancer Patients?

**DOI:** 10.1097/MD.0000000000003463

**Published:** 2016-05-06

**Authors:** In Ja Park, Chang Sik Yu, Seok-Byung Lim, Jong Lyul Lee, Chan Wook Kim, Yong Sik Yoon, Seong Ho Park, Jin Cheon Kim

**Affiliations:** From the Department of Colon and Rectal Surgery (IJP, CSY, S-BL, JLL, CWK, YSY, JCK); and Department of Radiology (SHP), University of Ulsan College of Medicine and Asan Medical Center, Seoul, Korea.

## Abstract

The present study explored the benefit of preoperative chemoradiotherapy (PCRT) for sphincter preservation in locally advanced low-lying rectal cancer patients who underwent stapled anastomosis, especially in those with deep and narrow pelvises determined by magnetic resonance imaging.

Patients with locally advanced low-lying rectal cancer (≤5 cm from the anal verge) who underwent stapled anastomosis were included. Patients were categorized into two groups (PCRT+ vs. PCRT–) according to PCRT application. Patients in the PCRT+ group were matched to those in the PCRT– group according to potential confounding factors (age, gender, clinical stage, and body mass index) for sphincter preservation. Sphincter preservation, permanent stoma, and anastomosis-related complications were compared between the groups. Pelvic magnetic resonance imaging was used to measure 12 dimensions representing pelvic cavity depth and width with which deep and narrow pelvis was defined. The impact of PCRT on sphincter preservation and permanent stoma in pelvic dimensions defined as deep and narrow pelvis was evaluated, and factors associated with sphincter preservation and permanent stoma were analyzed.

One hundred sixty-six patients were one-to-one matched between the PCRT+ and PCRT− groups. Overall, sphincter-saving surgery was performed in 66.3% and the rates were not different between the 2 groups. Anastomotic complications and permanent stoma occurred nonsignificantly more frequently in the PCRT+ group. PCRT was not associated with higher rate of sphincter preservation in all pelvic dimensions defined as deep and narrow pelvis, while PCRT was related to higher rate of permanent stoma in shorter transverse diameter and interspinous distance. On logistic regression analysis, PCRT was not shown to influence both sphincter preservation and permanent stoma, while longer transverse diameter and interspinous distance were associated with lower rate of permanent stoma.

PCRT had no beneficial effect on sphincter preservation in patients with locally advanced low-lying rectal cancer who had undergone stapled anastomosis. In patients with deep and narrow pelvis, PCRT had no impact on sphincter preservation but was associated with higher rate of permanent stoma.

## Abstract 2

Supplemental Digital Content is available in the text

## INTRODUCTION

It is generally expected that patients who require abdominoperineal resection would be able to preserve the sphincter via preoperative chemoradiotherapy (PCRT). Previous studies suggest that tumor downstaging and shrinkage after PCRT could increase the likelihood of sphincter preservation in rectal cancer patients.^1,2^

Many researchers have, however, reported controversial results regarding the influence of PCRT on sphincter preservation.^1–6^ Although many personal series reported an increased sphincter preservation rate after PCRT,^4–6^ 2 representative randomized controlled trials showed contrary results in terms of sphincter preservation following application of PCRT.^1,3^ Additionally, meta-analyses of randomized trials do not support increased rates of sphincter preservation after PCRT. A review of randomized trials revealed no difference in the rates of sphincter preservation between patients with and without PCRT.^6,7^ Similarly, a recent Cochrane review of 6 randomized trials found no positive effect of PCRT on the rate of sphincter preservation.^8^

Although the data do not strongly support the definite benefit of PCRT for sphincter preservation, clinicians and patients still expect that application of PCRT would increase the chance of sphincter preservation by shrinkage of tumor. Sphincter preservation is, however, a very complex issue involving tumor stage and location, the patient's habitus and wishes, and the surgeon's experience. Besides a direct effect of PCRT on tumor shrinkage, these factors would also be associated with sphincter preservation. It is assumed that the beneficial effect of PCRT on sphincter preservation would be much anticipated in more technically challenging situations, such as low-lying rectal cancer undergoing stapled anastomosis or anatomically difficult cases like deep and narrow pelvis.

Some studies have evaluated the operative difficulty of rectal cancer surgery according to the pelvic dimensions obtained using magnetic resonance imaging (MRI).^9,10^ In these reports, various pelvic dimensions were used to determine the depth and width of the pelvis. Combinations of these parameters could identify deep and narrow pelvises that could complicate sphincter preservation; however, the association of PCRT with sphincter preservation according to different pelvic dimensions was not evaluated.

In our present study, we investigated the impact of PCRT on sphincter preservation in patients with locally advanced low-lying rectal cancer who had undergone stapled anastomosis, and especially in those with deep and narrow pelvises determined by MRI.

## METHODS

### Patient Identification and Definition of Outcomes

Patients with locally advanced low-lying rectal cancer who were treated with radical resection between January 2009 and December 2012 were selected from the tumor registry of Asan Medical Center, Seoul, Korea. Patients who underwent stapled anastomosis were included. Locally advanced rectal cancer was defined as patients who were diagnosed as cT3-4 or node positive using MRI. Location of tumor was measured as distance from anal verge to lowest margin of tumor using digital rectal examination or rigid proctoscope. Low-lying rectal cancer was defined as low margin of tumor located below 5 cm from anal verge. Patients were excluded if they underwent local excision or observation after completion of PCRT or if they had manual coloanal anastomosis or intersphincteric resection.

Permanent stoma was defined as stoma persisting at least 2 years after the primary tumor resection. Anastomosis-related problems such as stricture, fistula, leakage, and abscess which required medical or surgical treatment were defined as anastomotic complications.

The database of Asan Medical Center was queried to identify clinicopathological and demographic characteristics. This study was approved by the institutional review board and informed consent was waived.

### Patient Categorization and One-to-One Match

Patients were categorized into 2 groups according to application of PCRT. Patients who received PCRT were classified as the PCRT+ group and those who did not receive PCRT were classified as the PCRT– group.

We made one-to-one matched group among the included patients of PCRT+ and PCRT– groups. Matched variables were age, sex, clinical T/N stage, and body mass index. Eighty three patients in each group were selected.

### Treatment, Pathologic Evaluation

Patients in the PCRT+ group received external beam radiation therapy (median dose, 50.4 Gy). Intravenous fluorouracil-based chemotherapy or capecitabine was given as concomitant chemotherapy. At 6 to 8 weeks after PCRT completion, patients underwent radical resection (low anterior resection or abdominoperineal resection) according to the principles of total mesorectal excision. Total mesorectal excision was performed in the PCRT– group without preoperative treatment and adjuvant treatment was delivered according to pathologic results.

In patients who received PCRT, pathologic responses to PCRT were evaluated in the resected specimens using the tumor regression grade scored using a 5-tier system: TR, total regression with no residual tumor cells and only fibrotic mass; NTR, near-total regression with microscopic residual tumor (i.e., difficult to find) in the fibrotic tissue; moderate regression, dominant irradiation-related changes with residual tumor (i.e., easy to find); minimal regression, dominant tumor mass with obvious irradiation-related changes; and no regression and no evidence of irradiation-related changes (fibrosis, necrosis, and vascular change). Patients with TR and NTR were considered good responder and others as nonresponder.

### Pelvic Dimensions on MRI

**FIGURE 1:**
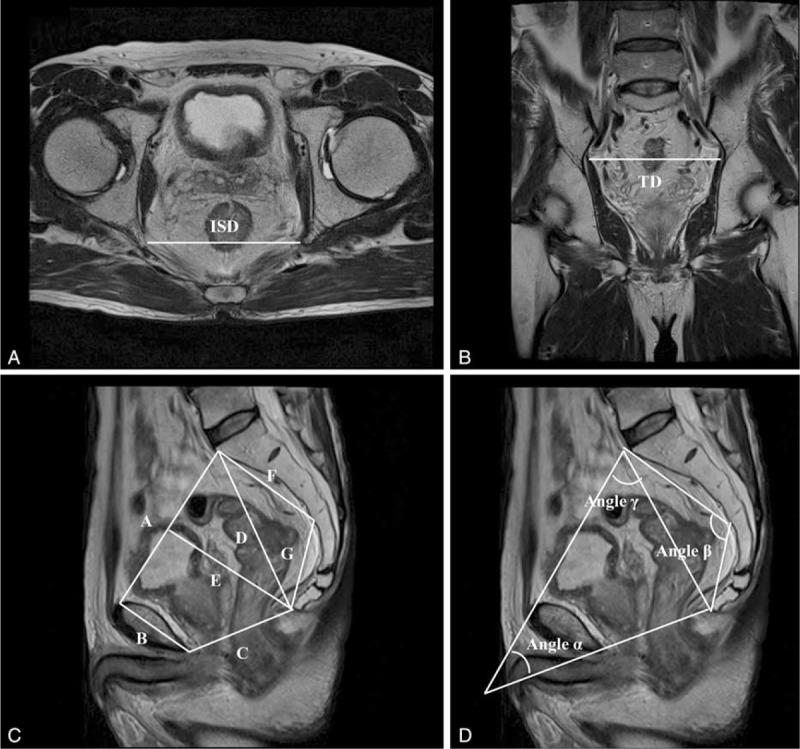
Pelvic dimension measurements in an (A) axial view, (B) coronal view, and (C, D) oblique view of pelvic magnetic resonance imaging.

Based on the preoperative staging MRI of the rectum, various pelvic dimensions were obtained. MRI was performed using a 1.5-T system (Magnetom Avanto; Siemens Medical Solutions, Erlangen, Germany); a dedicated 6-channel body phased-array coil was applied to the anterior side of the patient and another 6-channel spine coil was applied to the posterior side. The axial, sagittal, and oblique T2-weighted images of the pelvis were obtained using the following parameters: a 17 to 30 cm field of view, 3 to 4 mm section thickness, no intersection gap, 4100 to 6870 ms repetition time, 80 to 90 ms echo time, 220 × 256 matrix, and 117 echo train length. We considered various measurements of pelvic dimension as anatomical parameters that could influence sphincter preservation. Pelvic dimensions were obtained using mid-sagittal, axial, and oblique sections of the pelvis and 12 pelvic dimensions were measured (Table 1, Figure 1). Each pelvic dimension was categorized as deep and narrow pelvis when each value of pelvic dimension was lower than the median, except for angle α and *D* (pubic tubercle height) in which values higher than the median were considered deep and narrow pelvis.

**TABLE 1:**
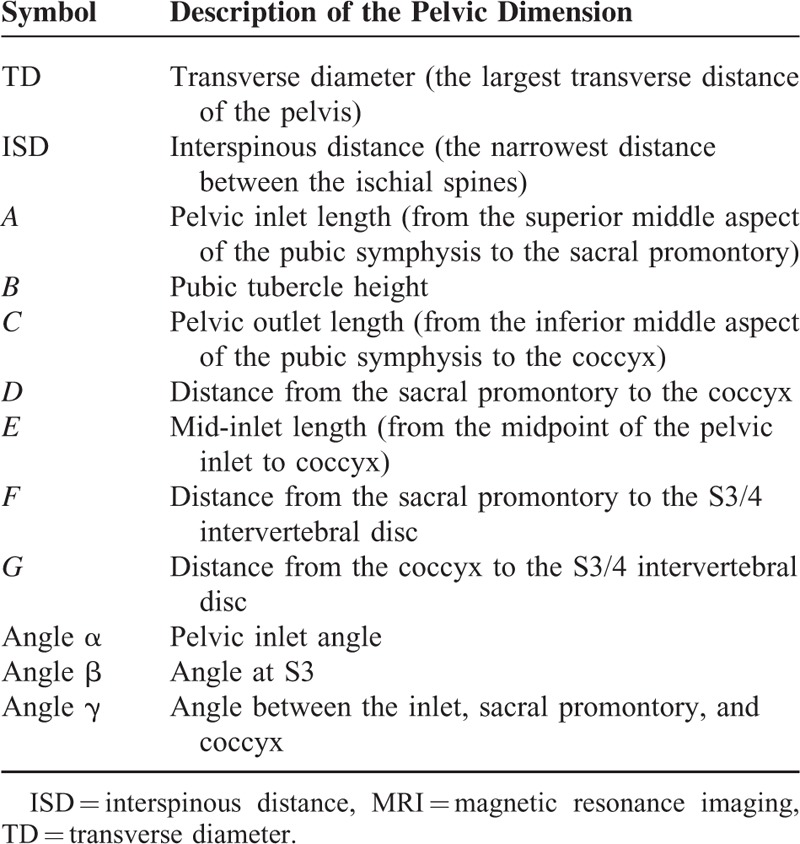
Pelvic Dimensions Measured on Axial, Sagittal, and Oblique MRI

### Outline of the Study and Statistical Analysis

We compared demographic features and surgical outcome between PCRT+ and PCRT–. Also, the rates of sphincter preservation, permanent stoma, and anastomotic complications were compared between the 2 groups. In the next part, the association between PCRT and sphincter preservation/permanent stoma in pelvic dimensions defined as deep and narrow pelvis was analyzed. Finally, multivariate analysis was performed to evaluate factors associated with sphincter preservation and permanent stoma.

Nonparametric data were compared using the Wilcoxon rank sum test. Categorical data were summarized by frequency within each group, and comparisons were made using the *χ*^2^ test for proportions. Univariate analyses were conducted to identify factors associated with sphincter preservation, including pelvic dimensions, and variables with *P* ≤ 0.1 in univariate analyses were included in multivariate logistic regression to identify factors independently and significantly associated with sphincter-preserving surgery performance. Values of *P* ≤0.05 were considered statistically significant. All statistical analyses were performed using SPSS (version 21.0; IBM Statistics, Armonk, NY).

## RESULTS

### Characteristics of the Study Patients

A total of 166 patients were included in the present study. The mean age was 57.85 years (range, 29–77 years). Men were more common in this population (n = 100, 60.2%). The mean body mass index was 23.47 kg/m^2^ (range, 16.52–34.7 kg/m^2^) and 46 patients (27.7%) had a body mass index higher than 25 kg/m^2^. The mean distance from the anal verge was 3.7 cm (range, 1–5 cm). The distance from the anal verge and the length of the distal resection margin were also comparable between the 2 groups. In the PCRT+ group, more than half of the included patients showed a good response to PCRT (Table 2). The pelvic dimension measurements were not significantly different between the 2 groups (Supplementary Table 1).

**TABLE 2:**
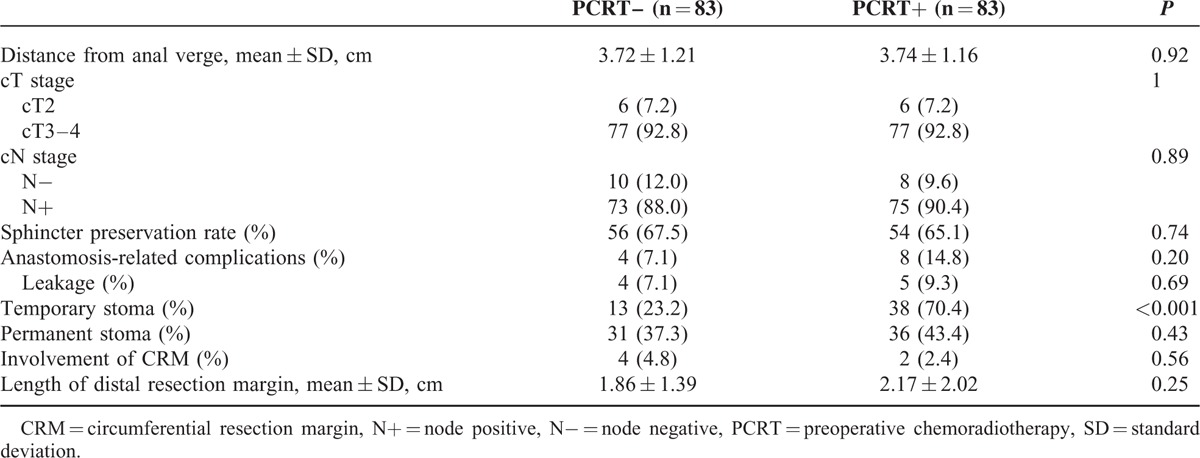
Patient Characteristics

### Sphincter Preservation, Stoma Formation, and Anastomotic Complications

The sphincter preservation rate was 66.3% overall, and the rates were not different between PCRT+ and PCRT– groups. Diverting stoma were made more frequently in the PCRT+ group and anastomosis-related complications, including leakage, also occurred more frequently in the PCRT+ group; however, the differences were not statistically significant compared with the PCRT– group. Reversal of diverting stoma was performed at 3 to 6 months after surgery. However, 7 patients of the PCRT+ group and 4 of the PCRT– group could not undergo reversal of diverting stoma or had to receive a stoma again after reversal because of anastomotic complications. In the PCRT+ group, 4 patients with anastomotic stenosis, 3 patients with recurrent fistula formation, and 1 patient with local recurrence could not reverse diverting stoma. In the PCRT– group, 1 patient maintained diverting colostomy due to a recurrent fistula and inflammation and the other patient with local recurrence underwent abdominoperineal resection. At the last follow-up, 36 patients in the PCRT+ group and 31 patients in the PCRT– group had permanent stoma (*P* = 0.43).

### Impact of PCRT on Sphincter Preservation and Permanent Stoma in Deep and Narrow Pelvis

For each pelvic dimension, the rates of sphincter preservation and permanent stoma were compared between the PCRT+ and PCRT– groups to evaluate an impact of PCRT on sphincter preservation or permanent stoma in any one of the anatomical features defined as deep and narrow pelvis (Table 3). As a result, PCRT was not associated with sphincter preservation in all pelvic dimensions. However, in patients with shorter than median transverse diameter (TD), the PCRT+ group had significantly more patients with permanent stoma than the PCRT– group (*P* = 0.05). The PCRT+ group also had a slightly higher permanent stoma rate than the PCRT– group in patients with shorter than median interspinous distance (ISD), but the difference was only marginally significant (*P* = 0.07).

**TABLE 3:**
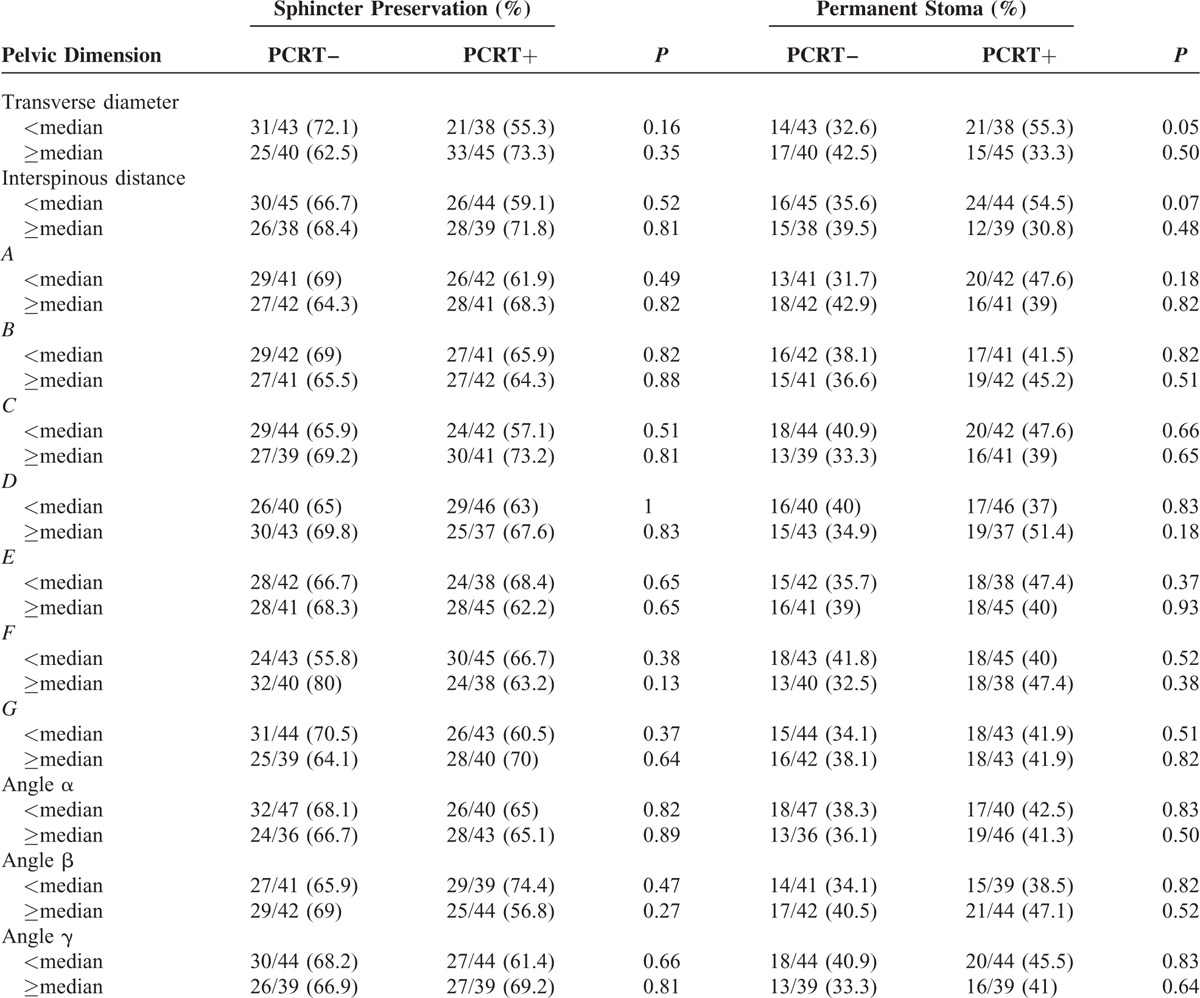
Association of PCRT Application With Sphincter Preservation and Permanent Stoma in Each Pelvic Dimension Defined as Deep and Narrow Pelvis

### Factors Associated With Sphincter Preservation and Permanent Stoma

We performed univariate and multivariate analysis to evaluate factors associated with sphincter preservation and permanent stoma, in patients with locally advanced, low-lying rectal cancer who underwent stapled anastomosis. The factors included in multivariate analysis were application of PCRT, response to PCRT, and the pelvic dimensions (TD and ISD) that showed significant association with permanent stoma in univariate analysis. In multivariate analysis, PCRT was not associated with sphincter preservation. In addition, longer TD and ISD were associated with decreased permanent stoma. The pathologic results of nonresponder to PCRT (moderate, minimal, and no regression) were associated with higher rate of permanent stoma and lower rate of sphincter preservation in the PCRT+ group, but only with marginal significance (Table 4).

**TABLE 4:**
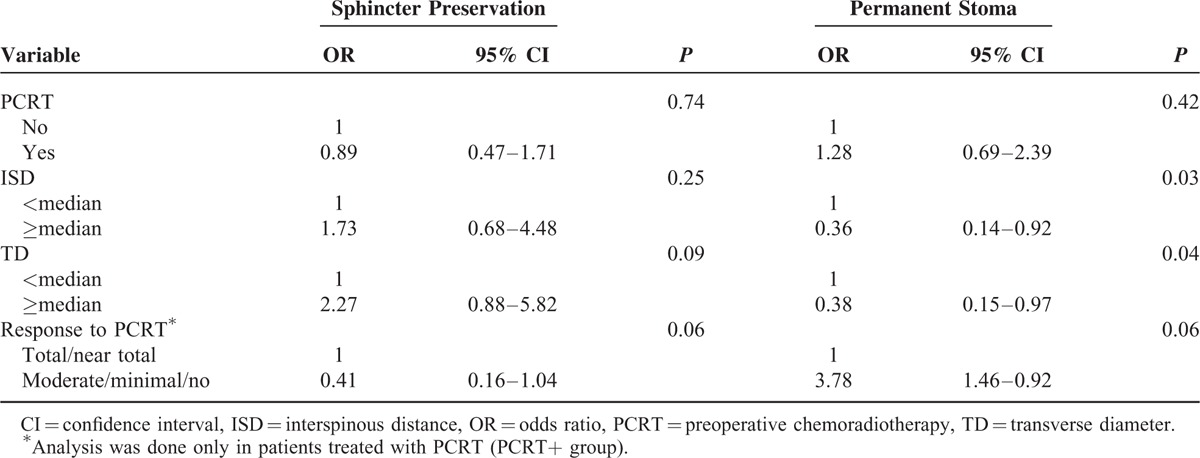
Factors Associated With Sphincter Preservation and Permanent Stoma

## DISCUSSION

In our present study, we showed that PCRT was not associated with increased sphincter preservation using stapled anastomosis in patients with locally advanced low-lying rectal cancer within a narrow and deep pelvis when patients were one-to-one matched for age, gender, body mass index, and clinical stage between groups classified according to application PCRT.

A previous German trial did show a 20% increase in the rate of sphincter preservation in a subgroup of patients who were determined to require PCRT before abdominoperineal resection prior to randomization,^1^ although sphincter preservation was not found to be different in the overall study population. The NSABP R-03 trial showed that the sphincter preservation rate did not differ between PCRT and postoperative CRT.^3^ Some studies have investigated whether the sphincter preservation rate could be increased by modification of the PCRT protocol, such as the radiation dose or concomitant chemotherapeutic regimen.^2,11,12^ The Lyon R96-02 trial^2^ suggested that high-dose preoperative radiotherapy could increase the rate of sphincter preservation compared with a conventional dose by increasing the clinical response to preoperative radiotherapy in patients with low-lying rectal cancer. However, the oncologic outcomes, including disease-free survival and overall survival, were similar between the 2 groups. Two other randomized trials could not find a benefit from PCRT in terms of sphincter preservation. The French ACCORD (Action Concertée cancer COloRectal et Digestif) 12 trial,^11^ despite a trend toward an increased rate of pathological complete response, showed no significant difference in the sphincter-preservation rate between a 45 Gy/concurrent capecitabine regimen (73%) and a 50 Gy/concurrent capecitabine + oxaliplatin regimen (75%). The Italian Studio Terapia Adiuvante Retto trial,^12^ which compared 2 neoadjuvant regimens according to the addition of oxaliplatin to 50.4 Gy with concurrent fluorouracil, found a similar rate of sphincter-saving procedures between the 2 arms. Based on the results of these studies, there is no definite evidence that PCRT improves sphincter preservation with current standard long-course PCRT.

Sphincter preservation is usually more challenging in low-lying rectal cancer than in mid- and upper rectal cancer. Therefore, PCRT is expected to play a beneficial role in sphincter preservation in low-lying rectal cancer. Although the subgroup analysis by Sauer et al^1^ was performed for patients who were preoperatively judged suitable for PCRT, many studies evaluating the effect of PCRT on sphincter preservation included all types of rectal cancer.^1,2,4–6^ Therefore, the effect of PCRT on sphincter preservation in challenging conditions has not been elucidated.

Sphincter preservation is a complex issue in rectal cancer patients because it depends on many factors, including tumor characteristics, patient age, sex, body habitus, patient psychology, and the surgeon's experience.^13–15^ Usually, physicians expect PCRT-induced tumor shrinkage to be beneficial for sphincter preservation in patients with anatomical features that make operations for rectal cancer difficult. Tumor shrinkage may facilitate the tumor to retreat from the anal verge and secure operative space by reducing tumor bulk.^16^

In the present study, the primary goal was to evaluate if there is an impact of PCRT on sphincter preservation in locally advanced low-lying rectal cancer patients, especially in those with deep and narrow pelvises. We attempted to control for factors that are considered to affect sphincter preservation, by matching the patients according to age, sex, clinical tumor stage, and body mass index. As a result, we found out that in patients with deep and narrow pelvis, as represented by 2 pelvic dimensions (shorter than median TD and ISD), PCRT was not associated with sphincter preservation but was associated with higher rate of permanent stoma. In the final part of our analysis where multivariate analysis was performed to evaluate factors associated with sphincter preservation and permanent stoma, deep and narrow pelvis was a factor associated with permanent stoma, but not with sphincter preservation.

The fact that PCRT was associated with higher rate of permanent stoma in certain pelvic dimensions representing deep and narrow pelvis, might have been caused by a high rate of anastomosis-related complications in the PCRT+ group. Indeed, the PCRT+ group showed 2 times higher anastomosis-related complication rate than the PCRT– group.

Poor tumor responses after PCRT were reported as predictive factors for the performance of abdominoperineal resection in previous studies, although this association is still controversial.^13–15^ In our present study, tumor regression grade assessed after PCRT was found to be associated with both sphincter preservation and permanent stoma, with marginal significance. Although further studies are needed in a larger number of patients to confirm these results, tumor regression grade after PCRT may provide practical information regarding prognosis after surgery.

Our present study included only the cases who had undergone stapled anastomosis. Although stapled anastomosis is clearly not superior to hand-sewn anastomosis for coloanal anastomosis, it may be preferred if an adequate distal margin can be attained because it is associated with improved function and shorter operative time.^17^ It is hard to apply a stapling device in patients with low-lying bulky rectal tumors in a deep and narrow pelvis but tumor shrinkage after PCRT may facilitate stapled anastomosis in these patients, and PCRT would have a practical benefit on sphincter preservation because hand-sewn coloanal anastomosis would be less influenced by tumor bulk or pelvic anatomy than stapled anastomosis. Although earlier studies have suggested that PCRT could facilitate anastomoses for low-lying tumors, few reports have examined the effects of PCRT in a certain anastomotic method used.^7,18^ One study has shown that PCRT tends to increase the possibility of the performance of double-stapled anastomosis for distal rectal cancer,^16^ in contrast to our present findings. We found that PCRT could not increase sphincter preservation using stapled anastomosis in locally advanced low-lying rectal cancer, both in general and specifically in patients with an unfavorable pelvic dimension measured by MRI. However, the rate of permanent stoma was increased in our current PCRT group in case of deep and narrow pelvis. It may be that immoderate stapling after PCRT can result in anastomotic complications that subsequently lead to permanent stoma. PCRT has been reported to be a risk factor of anastomotic complications^19,20^ and our present investigation revealed a higher level of anastomotic complications in the PCRT group. These results may be of practical assistance when we make surgical decision for patients who have locally advanced low-lying rectal cancer within a deep and narrow pelvis, and had undergone PCRT, that stapled anastomosis for such patients should be carefully considered since this surgical procedure was shown to carry risk for anastomotic complications and permanent stoma.

Our current study had some limitations of note. The variability in the measurement of the pelvic dimensions with pelvic MRI could have affected the outcomes, but we attempted to mitigate the interpretational bias through a central review of pelvic MRI results. Also, the fact that we included only stapled anastomosis cases in our study is another limitation. Therefore, sphincter preservation after PCRT for local excision, “wait-and-see,” and hand-sewn coloanal anastomosis approaches were not considered and the overall effect of PCRT on sphincter preservation would thus have been underestimated by our analysis. In addition, we did not include functional data, which represents the quality of life that is achieved by sphincter preservation, or qualified measurements of successful sphincter preservation.

Our present study did however evaluate the effect of PCRT on sphincter preservation using stapled anastomosis according to pelvic dimensions that may influence operative difficulty. In patients with deep and narrow pelvis, we found that PCRT could not facilitate stapling anastomosis in terms of preserving sphincter function and actually interfered with stoma reversal. Our current findings thus provide practical information for the planning of operative procedure. Faced with an unfavorable pelvic anatomy, PCRT should be carefully considered for the purpose of sphincter preservation in locally advanced low-lying rectal cancer patients who undergo stapled anastomosis.

In conclusion, PCRT did not show a beneficial effect on sphincter preservation following stapled anastomosis in patients with locally advanced low-lying rectal cancer in deep and narrow pelvis. However, other surgical methods such as local excision or wait-and-see approaches were not included in our present analyses. Therefore, the results of this study are not representative of the overall influence of PCRT on sphincter preservation in rectal cancer patients. Further studies that include diverse surgical options after PCRT could help to verify the overall effect of PCRT on sphincter preservation.

## Supplementary Material

Supplemental Digital Content
