# Supplementary material for: Is Preoperative Chemoradiotherapy Beneficial for Sphincter Preservation in Low-Lying Rectal Cancer Patients?
Source: Medicine (Baltimore). 2016 May 6;95(18):e3463. doi: 10.1097/MD.0000000000003463 (PMC4863762; doi:10.1097/MD.0000000000003463)
Supplement: Supplemental Digital Content [file medi-95-e3463-s001.docx]

Supplementary Table 1. Pelvic dimensions according to PCRT administration.

| Pelvimetric parameter | PCRT– | PCRT+ | *p* |
| --- | --- | --- | --- |
| Transverse diameter | 11.23 ± 0.83 | 11.37 ± 0.80 | 0.29 |
| Interspinous distance | 9.32 ± 1.04 | 9.27 ± 0.6 | 0.75 |
| A | 11.59 ± 1.05 | 11.52 ± 0.92 | 0.62 |
| B | 5.08 ± 0.41 | 5.13 ± 0.39 | 0.49 |
| C | 8.21 ± 0.70 | 8.22 ± 0.70 | 0.96 |
| D | 12.27 ± 1.32 | 12.23 ± 1.19 | 0.86 |
| E | 10.83 ± 0.96 | 10.71 ± 0.91 | 0.40 |
| F | 7.92 ± 0.67 | 7.92 ± 0.66 | 0.97 |
| G | 6.42 ± 0.79 | 6.41 ± 0.81 | 0.89 |
| Angle α | 45.63 ± 5.08 | 47.0 ± 5.21 | 0.09 |
| Angle β | 117.82 ± 10.40 | 117.54 ± 9.12 | 0.85 |
| Angle γ | 61.86 ± 6.53 | 61.88 ± 6.16 | 0.98 |

All data are the mean ± standard deviation.
